# Examining Physical and Social Environments of Korean American Family Caregivers of Persons Living with Dementia

**DOI:** 10.1093/geroni/igab046.2954

**Published:** 2021-12-17

**Authors:** Kathy Lee, Chang Hyun Seo, Jon Shuffler, Christina Miyawaki, Joshua Grill, Hye-Won Shin

**Affiliations:** 1 University of Texas at Arlington, Coppell, Texas, United States; 2 University of Texas at Arlington, University of Texas at Arlington, Texas, United States; 3 University of Houston, University of Houston, Texas, United States; 4 UC Irvine Institute for Memory Impairments and Neurological Disorders (UCI MIND), UC Irvine Institute for Memory Impairments and Neurological Disorders (UCI MIND), California, United States

## Abstract

**Introduction:**

East Asian Americans are considered a hard-to-reach population in the field. Existing resources are not inclusive of Korean family caregivers, and therefore, a community support system may be required for this ethnic group.

**Methods:**

We conducted a telephone-based survey and in-depth interview with Korean family caregivers of persons with dementia (N=36; Mean age: 63.3) to examine their physical and social environments.

**Results:**

Most caregivers (58%) co-resided with their care recipients. Those providing care with limited assistance from others showed greater financial hardship (p=0.03) and interference with employment (p=0.03). Job interference was further related to higher levels of caregiving burdens (p=0.01). The services used most were senior center services (25%) and in-home care services (17%), while desired services included health promotion and disease prevention services (50%) and culturally appropriate or medically tailored home-delivered meals services (31%). Caregivers socialized with others using a multi-messaging app (i.e., KakaoTalk) with others. About 74% of them addressed they used KakaoTalk always (52%) or often (22%), and nearly half of them (47%) said they searched for caregiving information online. Findings from our qualitative interviews confirmed positive attitude toward the use of technology. Korean family caregivers showed a lack of knowledge of not only existing community-based resources but also the disease-related information, particularly regarding early-stage support and home safety.

**Conclusion:**

It is critical to develop a community education program that reflects their unique physical and social environment conditions, potentially through technologically delivered interventions, for outreach and engagement for Korean family caregivers of persons with dementia.

